# Editorial and Miscellaneous

**Published:** 1864-04

**Authors:** 


C. S. Medical & Surgical Journal.

RICHMOND, APRIL, 1864.

*AYRES & WADE......PUBLISHERS AND PROPRIETORS.*

### *Artificial Limbs.—How to Make Them.*

The establishment of an association to provide, gratuitously, artificial limbs to all officers, soldiers, or sailors, who have been maimed in the service of the Confederate States, has been already noticed in these columns. The fact of its successful operation should not be without its effect on operative surgery in the future.

Up to this stage in the war, the principal and paramount consideration with each conscientious surgeon, who amputated a limb, was that great deformity, or a speedy and miserable death, could only be prevented by this mutilation. The possibility of the early adjustment of an artificial substitute for the limb by a skillful mechanician was not then realized as it will be in future, and will now compel the surgeon to look beyond the operation and recovery from the wound he makes. Already, nearly ten thousand maimed men in the Confederate States carry with them stumps which will be examined soon by those specialists, who are experts in this limited field of anatomy and mechanical philosophy. The stumps will be all sure but silent witnesses of skill and watchfulness, or inexperience and neglect.

These considerations should cause more deliberate attention to be paid to the place of operation, whether of election or necessity, the various methods of amputation, the progressive steps of each, and the natural or possible changes in the several structures as bearing on the desired result, viz: a *successful stump*, or one that will enable the patient to wear an artificial limb with comfort or convenience. Its uses being for locomotion and the bearing of pressure, a good stump should, as far as possible, be long enough to secure abundant leverage, be well covered, perfectly even, and of the shape of a truncated cone, to equally diffuse the pressure and support over the entire circumference. The socket of the artificial limb, being well padded, is made to fit this stump in every portion of its periphery, the extremity being free in the interior cavity. The places of election are authoritatively pointed out in operative surgeries, but I have seen no clearer or more comprehensive instructions on this or kindred subjects than those published by Palmer, of Philadelphia, in "Instructions to operators for formation of suitable stumps in amputation of the leg and thigh." The surgeon, unfortunately, rarely has this power of election, and in such circumstances, must leave as much substance as he can.

Artificial limbs resemble those of the *insecta* in that the skeleton is without. Their structure is generally of wood, covered with leather, but sometimes of heavy wire-cloth. Strong and well-secured joints at points corresponding with those in the lost limb, move with ease and exactness through the medium of cords, levers and springs of metal or gutta-percha, the whole being in symmetry and function a faithful representation of the natural leg, with the exception that the artificial leg, being but the work of man, is a more simple apparatus. The complex parts and functions of the natural leg could not be *so* constructed and co-ordinated.

Palmer, of Philadelphia, obtained the prize medal at the World's Fair, in London, in 1850, for the best artificial leg. The Academy of Medicine, of New York city, in 1860, after a careful examination of specimens of all well known varieties, pronounced one made and patented by Dr. Bly, of Rochester, as quite as simple as any, and superior to all in possessing valuable improvements in the lateral motion given the ankle-joint. Among those there inspected were Selpho's, Wilcox's, Ord's and Jewett's legs. The artificial leg found on the body of Colonel Ulric Dahlgren, U. S. A., was of Jewett's patent, January 6th, 1857, and May 7th, 1860. Its construction was very simple, but its weight too great. Its substance is wood, painted flesh color, and heavily enamelled to resemble porcelain; the metal joints were silver-plated and very strong. Thus its symmetry and finish gave it an appearance of elegance; but its mechanism and action are inferior to that of legs manufactured under some other patents.

These legs can be made very easily by any good locksmith, gunsmith, instrument-maker, or ingenious mechanic, and good specimens are now being made at various points in the Confederate States, by persons recently inexperienced. Others, as Hanger, of Staunton, and Wells, of Charlottesville, claim to have made valuable improvements on any hitherto known patent. They certainly make legs combining lightness, strength and symmetry, that are worn with comfort and satisfaction by officers and men in the field and in every station of life, civil and military.

But the manufacturers now in operation are not able to fill one-tenth of the demand that will be made on the association. To meet them, a manufactory on a large scale will be established, and the directors of the association referred to have also invited manufacturers, throughout the Confederacy, to send in to them, at Richmond, Va., as soon as practicable, specimens of their work, with proposals, stating the number they can furnish, and the time and place of delivery, with the cost of the same.

The United States Patent Office Reports, in every large library in the Confederate States, contain diagrams of all patents of artificial legs and their description, and an experienced draughtsman in Richmond (Mr. H. M. Baker) will furnish copies of all of them, at a moderate cost, to any one proposing to commence their manufacture.

